# Follow-Ups on Persistent Symptoms and Pulmonary Function Among Post-Acute COVID-19 Patients: A Systematic Review and Meta-Analysis

**DOI:** 10.3389/fmed.2021.702635

**Published:** 2021-09-03

**Authors:** Qiuyue Long, Jiwei Li, Xiaoyi Hu, Yangyuyan Bai, Yali Zheng, Zhancheng Gao

**Affiliations:** ^1^Department of Respiratory, Critical Care and Sleep Medicine, Xiang'an Hospital of Xiamen University, Xiamen, China; ^2^School of Medicine, Xiamen University, Xiamen, China; ^3^Department of Respiratory Medicine, Peking University People's Hospital, Beijing, China

**Keywords:** SARS-CoV-2, post-acute COVID-19 syndrome, post-discharge, epidemiology, meta-analysis

## Abstract

**Objective:** As the number of recovering COVID-19 patients increases worldwide, the persistence of symptoms and signs through the post-acute phase indicates an urgent need for prolonged follow-up care. To explore existing data about post-acute COVID-19 syndrome, this meta-analysis assesses the prevalence of persistent manifestations in multiple systems and abnormalities in lung function, as well as their related risks in patients with various severities.

**Methods:** Articles about discharged COVID-19 patients (published from January 1, 2020 to February 23, 2021) were obtained by searching four databases. Cohort studies with follow-up periods >1 month post-discharge or >2 months post-admission were included.

**Results:** A total of 4,478 COVID-19 patients from 16 cohort studies were included in this meta-analysis. Fatigue or weakness (47%) were the most prevalent physical effects of post-acute COVID-19 syndrome, while psychosocial (28%) symptoms were the most common manifestations among several systems. Abnormalities in lung function of recovering patients, i.e., DLCO <80% (47%, 95% CI: 32–61%) persisted for long periods. Severe patients were more likely to present joint pain (OR 1.84, 95% CI: 1.11–3.04) and decreased lung functions compared with non-severe patients, with pooled ORs for abnormal TLC, FEV1, FVC, and DLCO of 3.05 (95% CI: 1.88–4.96), 2.72 (95% CI: 1.31–5.63), 2.52 (95% CI: 1.28–4.98), and 1.82 (95% CI: 1.32–2.50), respectively.

**Conclusions:** Our research indicates that patients recovering from COVID-19 manifest long-term, multi-system symptoms, and the adverse effects on psychosocial health and lung functions were the most extensive and persistent. These findings together may facilitate much needed in-depth study of clinical treatments for long-term, post-acute phase symptoms that affect a great number of recovering COVID-19 patients.

## Introduction

Coronavirus disease 2019 (COVID-19) is a respiratory disease caused by a highly transmissible and pathogenic virus, the severe acute respiratory syndrome coronavirus 2 (SARS-CoV-2) (1). So far, more than 100 million people worldwide have contracted COVID-19. Recovery is gradual and it is estimated that most COVID-19 patients take an average of 2–6 weeks to recover. However, symptoms may persist for weeks or even months in a subset of patients even after their initial hospital discharge. In addition, some patients may even manifest medical complications or sequelae that adversely affect their long-term health (2, 3). This phenomenon has been described as “long COVID-19,” also known as “post-acute COVID-19 syndrome,” which is defined as persistent signs and symptoms that emerge during or after SARS-CoV-2 infection, usually lasting more than 4 weeks and with all other possible diagnoses excluded (4).

A clinical investigation (5) has found that COVID-19 survivors were more likely to develop medical sequelae than patients never infected with SARS-CoV-2, strongly suggesting that these signs and symptoms may indeed be adverse consequences of COVID-19. A recent study (6) reported that 32% of hospitalized COVID-19 patients presented with one to two symptoms at 2 months after disease onset, 55% presented three or more symptoms, while only 12.6% had no symptoms at all. In another study (7), more than 70% of patients still exhibited at least one symptom even at 6 months after onset. With the steady increase in patients recovering from COVID-19 globally, public focus has gradually shifted away from the rapid disease progression in the acute phase to the long-term health effects of COVID-19. In the meanwhile, there is an increasingly urgent need for clear guidelines regarding how to alleviate the burden of COVID-19 symptomatic aftershock.

Although lungs are the primary target of SARS-CoV-2 infection, evidence from the acute phase also has shown that extra-pulmonary manifestations of COVID-19 can occur in a surprisingly wide range of organs (8). The underlying pathophysiological mechanisms (9–11) include direct viral invasion, systemic inflammation, endothelial injury mediated by infection, as well as disorders of the immune system among others. Therefore, the adverse effects of post-acute COVID-19 may similarly involve multiple organ systems. Based on previous studies of Severe Acute Respiratory Syndrome (SARS) and Middle East Respiratory Syndrome (MERS) survivors (12, 13), several short-term and potential long-term outcomes have been proposed for SARS-CoV-2 (14). Specifically, the relevant adverse consequences mainly occur in the immune, respiratory, cardiovascular, neurological, and gastrointestinal systems as well as affecting mental health. Furthermore, a small proportion of patients with severe or critical diseases, especially those who have undergone ventilator support in an intensive care unit (ICU), were found to have a higher risk of developing organ-specific, functional, and cognitive disorders than those with less severe diseases (15). Currently, guidelines based on epidemiological evidence from other coronaviruses have been proposed for the rehabilitation of post-COVID-19 patients (16, 17). However, it is still necessary to further determine the indeed long-term adverse symptoms and functional abnormalities of the post-COVID-19 syndrome and explore closely related risk factors to establish targeted and effective intervention measures.

To this end, we performed a meta-analysis of cohort studies that described the residual symptoms and pulmonary function tests (PFT) of discharged COVID-19 patients (including those discharged from ICU) to determine the adverse effects on multiple systems and differences between severe and non-severe patients in the post-acute phase. Our findings will provide clinicians and physicists with a summary of information relevant to the rehabilitation, treatment, and management of post-acute COVID-19 patients, thus facilitating better preparedness and management of the long-term consequences of the global pandemic.

## Methods

This meta-analysis was conducted using the guidelines described in the Preferred Reporting Project of Systematic Review and Meta-Analysis (PRISMA) and registered in PROSPERO under study number CRD42021238955.

### Search Strategy and Selection Criteria

A systematic search was conducted on studies published in PubMed, Embase, Web of Science, and WHO COVID-19 Database from January 1, 2020 to February 23, 2021. Search terms including “COVID-19,” “SARS-CoV-2,” “2019-nCoV” and “novel coronavirus” were used to find articles related to the novel coronavirus pandemic. To obtain literature pertaining specifically to post-acute outcomes, the terms “long-term effect,” “post-acute,” “post-discharge,” “long-COVID,” and “chronic-COVID” were used to screen the hits obtained using the broader COVID-19 search terms. Only articles in English were considered for inclusion. To identify missing studies, we checked the bibliography of each selected paper. Records were managed by EndNote X9.0 software to exclude duplicates. Full-text screening was performed for publications describing systemic manifestations and pulmonary function of adult hospitalized COVID-19 patients. Only cohort studies were used for analysis. Studies were excluded for the following criteria: (a) they were reviews, preprints, comments, editorials, or case reports; (b) lacked data regarding primary outcomes; or (c) if they included outpatients or had insufficient follow-up exams (<1 month post-discharge or <2 monthspost-admission).

### Risk of Bias Assessment

The Newcastle-Ottawa scale (NOS) was used to assess the risk of bias in non-randomized cohort studies through eight criteria across three overall categories related to study rigor, including participant selection methodology, comparability with other studies, and evaluation of outcomes. Quality was assessed by scoring each publication with a star based on whether it used the most reliable reporting for each of eight criteria (with some criteria allowing multiple stars). The highest possible score was a full score of 9 stars. Cohort studies that received <6 stars, 6 to 7 stars, or more than 7 stars had a high, medium, or low risk of bias, respectively.

### Data Extraction and Definitions

The two reviewers (XH and YB) who performed the literature search also independently extracted the relevant data from the included studies. Disagreements were resolved with a third reviewer (QL) or by consensus. The following variables were extracted: study features (first author, year, country, study design), population characteristics (age, sex, sample sizes, follow-ups, severity groups), and outcomes (multi-system symptoms and lung functions). The diagnosis of COVID-19 was based on WHO guidelines and confirmed by reverse transcription-polymerase chain reaction (RT-PCR), next-generation sequencing, or chest imaging. Severe COVID-19 was self-defined by each of the included studies. For studies lacking a definition of disease severity, severe cases were defined as those with a WHO ordinal score of 5 (noninvasive ventilation or high flow oxygen therapy), 6 (intubation and mechanical ventilation), 7 (additional organic support), or patients admitted to the ICU. According to the included studies, symptoms other than fatigue or weakness were categorized as cardiopulmonary, neurological, gastrointestinal, musculoskeletal, psychosocial system, or other. Total lung capacity (TLC), diffusion capacity for carbon monoxide (DLCO), forced expiratory volume in the first second (FEV1) and forced vital capacity (FVC) <80% of the predicted value, as well as FEV1/FVC <70% were defined as abnormal lung functions.

### Data Analysis

Data were analyzed by Stata MP16.0 software (Stata Corp LP, College Station, Texas, USA). Symptoms and abnormal lung functions appeared in at least three studies that were included in the meta-analysis. The prevalence was calculated based on the occurrence of events, standard error (SE), and 95% confidence intervals (CI) in each study. Odds ratio (OR) was used to describe the risk of events in severe patients compared with non-severe patients. Heterogeneity was determined by Cochran's *Q*-test and *I*^2^ statistical analysis. A fixed-effect model was applied for studies with acceptable levels of heterogeneity (*I*^2^ <50%, *P* > 0.1). Otherwise, a random-effects model was used to adjust for significant heterogeneity. Sensitivity analysis was used to evaluate the robustness of the comprehensive results by re-analyzing the data in aggregate, independently of individual studies.

## Results

### Study Selection and Risk of Bias

**Figure 1:**
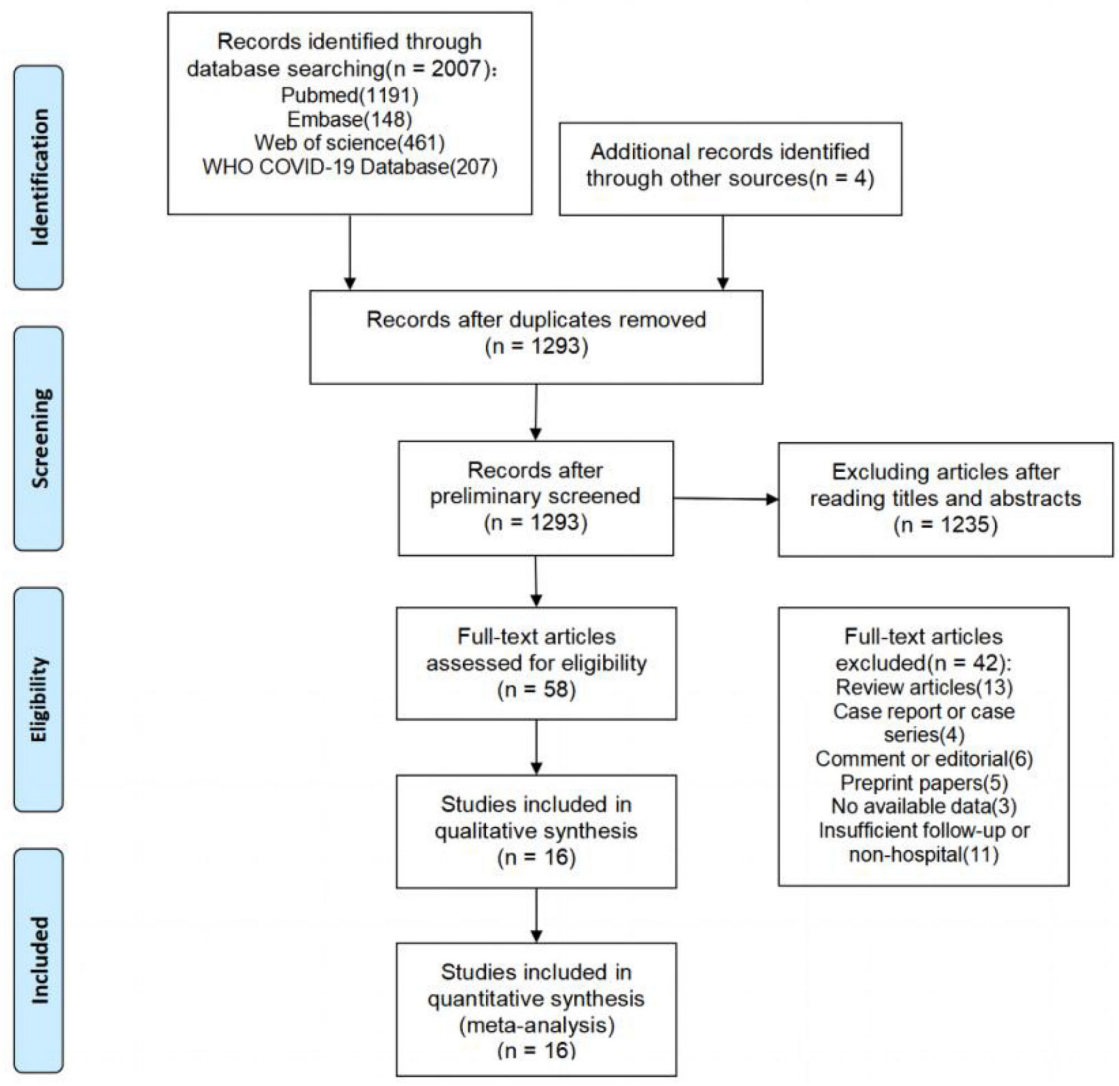
Flow chart of the number of studies screened and included in the meta-analysis.

The literature search yielded a total of 2,011 articles, 2,007 of which were obtained through database searches and the remaining four articles were identified through other search methods (Figure 1). Following de-duplication, 1,293 records were reviewed by screening titles and abstracts, of which 58 appeared to be eligible for this analysis. After full-text screening, 16 cohort studies (5, 7, 18–31) were found to meet the inclusion criteria, and were thus used in the meta-analysis. These studies were mainly from Europe, China, and the United States. Of the 16 included studies, only 2 (12.5%) were rated as high quality (≥7 stars) by NOS assessment, while the remaining studies were of moderate quality (ten studies with 6–7 stars; four studies with 5 stars), primarily due to poor comparability and/or outcome bias. Both post-acute symptoms and lung function indicators showed significant heterogeneity in our study. We therefore conducted sensitivity analysis and the results showed that no single publication affected the final pooled prevalence (see Supplementary Figures).

### Study Population Characteristics and Outcomes

The main characteristics of the included cohort studies are shown in Table 1. Most of the studies (10/12, 83.3%) included in this meta-analysis were prospective cohort studies. The combined total of 4,478 COVID-19 patients included in our analysis were diagnosed predominantly by RT-PCR, with sample sizes for each study ranging from 55 to 1,733 patients. Among them, 2,309 (51.56%) were men (median or mean ages generally between 50 and 60 years old). Study follow-ups lasted for 1–4 months post-discharge or 2–6 months post-admission. The manifestations of post-acute COVID-19 syndrome recorded in these studies included a wide range of multiple affected organs, involving the cardiopulmonary, neurological, musculoskeletal, gastrointestinal, and psychosocial systems (see Supplementary Table 1). The cardiopulmonary system had the widest spectrum of related symptoms, including chest pain, dyspnea, cough, sore throat, palpitation, and chest distress. Symptoms associated with the neurological system included memory impairment, cognitive impairment, headache, taste disorder, and smell disorder. Musculoskeletal symptoms included myalgia and joint pain. Gastrointestinal symptoms included diarrhea or vomiting, abdominal pain, and decreased appetite. Psychosocial manifestations included Post-Traumatic Stress Disorder (PTSD), anxiety or depression, attention deficit disorder, sleep difficulties, and hair loss. Fatigue or weakness, skin rash, fever, pain, discomfort, and dizziness, which were occasionally reported among discharged patients, were categorized into other symptoms. In addition, seven studies investigated abnormal lung function in post-acute patients. These studies included multiple parameters, such as DLCO for lung diffusion capacity, TLC for restrictive respiratory function, and FEV1 and FVC for obstructive respiratory function.

**Table 1:** Main characteristics of included studies in the meta-analysis.

**Author**	**Country**	**Study design**	**COVID-19 diagnosis**	**Sample sizes**	**Age**	**Sex (M %)**	**Follow-up time (days)**	**Settings**	**NOS assessment**
Huang C	China	Prospective cohort study	RT-PCR	1,733	Median 57·0 (IQR 47.0–65.0)	52%	PA:180	Scale 3 (439) Scale 4 (1,172) Scale 5–6 (122)	8
Qin W	China	Prospective cohort study	RT-PCR	647	Mean 58.0 (± 15.0)	44%	PD:90	Severe (248) Non-severe (399)	8
Sykes DL	UK	Prospective cohort study	RT-PCR	134	Median 58.0 (range 25.0–89.0)	65.7%	PD:113 (range 46–167)	Ward (107) ICU (27)	7
Garrigues E	France	Prospective cohort study	RT-PCR CT	120	Mean 63.2(± 15.7)	62.5%	PA:110.9 (± 11.1)	Ward (96) ICU (24)	7
van der Sar-van der Brugge S	Netherlands	Prospective cohort study	RT-PCR	101	Mean 66.4 (± 12.6)	57.4%	PD:42	Scale 3 (28) Scale 4 (73)	7
Jacobs LG	USA	Prospective cohort study	RT-PCR	183	Median 57.0 (IQR 48.0–68.0)	61.5%	PD:35 (± 5)	–	6
Arnold DT	UK	Prospective cohort study	RT-PCR	110	Median 60.0 (IQR 46.0–73.0)	56%	PD: 83 (IQR 74–88)	Mild (27) Moderate (65) Severe (18)	6
Bellan M	Italy	Prospective cohort study	RT-PCR	238	Median 61.0 (IQR 50.0–71.0)	59.7%	PD:120	–	6
Halpin SJ	UK	Prospective cohort study	RT-PCR	100	Median Ward 70.5 (range 20–93) ICU 58.5 (range 34–84)	54%	PD:48 (± 10.3)	Ward (68) ICU (32)	6
Suárez-Robles M	France	Prospective cohort study	RT-PCR	134	Mean 58.53 (± 18.53)	46.3%	PD:90	–	5
Méndez R	Spain	Prospective cohort study	RT-PCR	179	Median 57.0 (IQR 49–67)	41.3%	PD:60	–	5
Raman B	UK	Prospective cohort study	RT-PCR	58	Mean 55.4 (± 13.2)	58.6%	PA:60–90	–	5
Taboada M	Spain	Prospective cohort study	RT-PCR	91	Mean 65.5(± 10.4)	64.8%	PA:180	–	6
Xiong Q	China	Prospective cohort study	RT-PCR	538	Median 52.0 (IQR 41.0–62.0)	45.5%	PD: 97 (IQR 95–102)	General (331) Severe (180) Critical (27)	7
Zhao YM	China	Retrospective cohort study	RT-PCR	55	Mean 47.74 (± 15.49)	58.18%	PD:90	Mild (4) Moderate (47) Severe (4)	5
Huang Y	China	Retrospective cohort study	RT-PCR NGS	57	Mean 46.72 (± 13.78)	45.6%	PD:30	Severe (17) Non-severe (4)	6

*CT, computed tomography; RT-PCR, reverse transcription polymerase chain reaction; NGS, next generation sequencing; IQR, interquartile range; PA, post admission; PD, post discharge; ICU, intensive care unit*.

### Prevalence of Post-acute Manifestations in Multiple Organ Systems

As shown in Table 2, among several organ systems, psychosocial (28%, 95% CI 24–31%) manifestations were most common and the prevalence of its three symptoms including anxiety or depression, sleeping difficulty (27%, 95% CI 21–32%), and hair loss (24%, 95% CI 19–29%) was generally higher than most symptoms in other systems. Followed by cardiopulmonary (15%, 95% CI 13–17%) and neurological system (15%, 95% CI 12–19%), both had similar prevalence of overall symptoms. But the prevalence of their single symptom varied widely, especially the cardiopulmonary, ranging from 5 to 33%. The next was the musculoskeletal system (13%, 95% CI 9–16%), including myalgia (13%, 95% CI 8–18%) and joint pain (12%, 95% CI 8–16%). In contrast, the gastrointestinal (7%, 95% CI 4–10%) had the lowest prevalence of symptoms. Other symptoms such as fever (2%, 95% CI 0–3%) and skin rash (3%, 95% CI 1–5%), were even rarer. In general, the most prevalent post-acute COVID-19 symptom was fatigue or weakness (47%, 95% CI 36–59%), then the memory impairment (35%, 95% CI 21–48%) and anxiety or depression (33%, 95% CI 23–43%), dyspnea (33%, 95% CI 22–43%)followed.

**Table 2:** The prevalence of symptoms and manifestations in multiple systems.

**Outcomes**		**Studies**	**Effects**		
			**Prevalence (95% CI)**	***I*^**2**^**	***p* for heterogeneity**
Fatigue or weakness		9	47% (36–59%)	97%	<0.01
Cardiopulmonary	Sputum	3	7% (1–13%)	–	–
	Chest pain	8	7% (5–10%)	95.29%	<0.01
	Dyspnea	9	33% (22–43%)	97.83%	<0.01
	Cough	8	17% (11–22%)	95.01%	<0.01
	Sore throat	4	5% (3–8%)	76.1%	0.01
	Palpitation	4	11% (9–14%)	75.86%	0.01
	Overall		15% (13–17%)	96.99%	<0.01
Musculoskeletal	Myalgia	6	13% (8–18%)	95.66%	<0.01
	Joint pain	7	12% (8–16%)	88.46%	<0.01
	Overall		13% (9–16%)	95.22	<0.01
Neurological	Memory impairment	4	35% (21–48%)	91.25%	<0.01
	Headache	4	15% (3–26%)	95.56%	<0.01
	Taste disorder	6	10% (6–13%)	77.03%	<0.01
	Smell disorder	8	11% (8–14%)	80.59%	<0.01
	Overall		15% (12–19%)	96.04%%	<0.01
Gastrointestinal	Diarrhea or vomiting	5	3% (1–6%)	86.71%	<0.01
	Decreased appetite	3	14% (5–23%)	–	–
	Overall		7% (4–10%)	93.6%	<0.01
Psychosocial	Anxiety or depression	5	33% (23–43%)	92.18%	<0.01
	Sleep difficulties	6	27% (21–32%)	83.83%	<0.01
	Hair loss	3	24% (19–29%)	–	–
	Overall		28% (24–31%)	86.46%	<0.01
Other	Skin rash	3	3% (1–5%)	–	–
	Fever	4	2% (0–3%)	82.87%	<0.01
	Overall		2% (1–4%)	91.63%	<0.01

### Prevalence of Abnormal Lung Function

**Figure 2:**
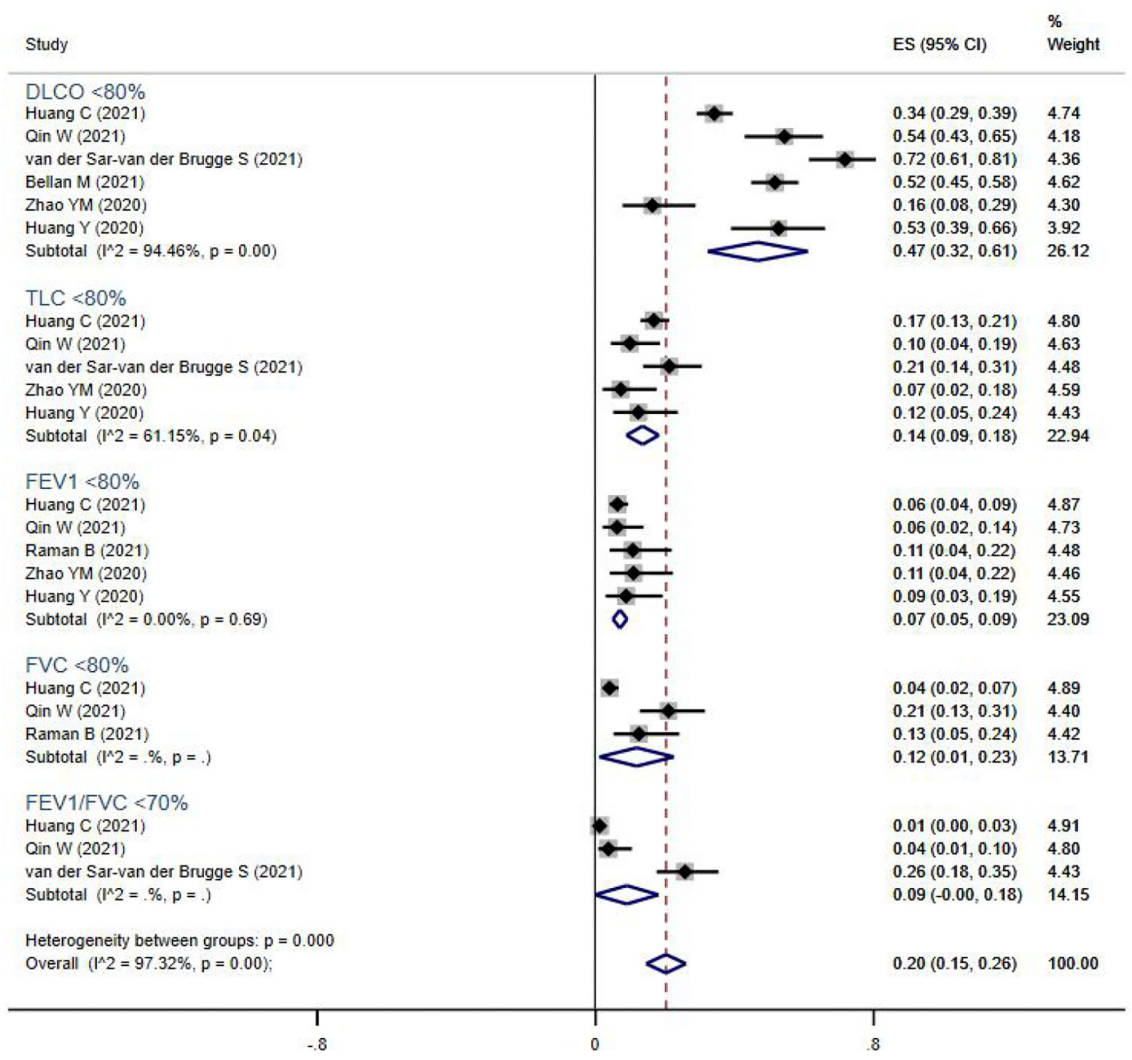
The forest plot of the prevalence of abnormal lung function parameters, with a range from 1 to 72%. The brown dashed line at 0.2 on the x-axis represents the overall prevalence (20%, 95% CI 15–26%) of these parameters. The blue rhombuses represent prevalence and 95% CI of each outcome. DLCO, diffusion capacity for carbon monoxide; TLC, total lung capacity; FEV1, forced expiratory volume in the first second; FVC, forced vital capacity.

Pulmonary function tests (including spirometry, lung volume, and diffusion capacities) were reported for 894 subjects from seven studies (Figure 2). The overall prevalence of abnormalities in lung function was 20% (95% CI 13–17%), and included low diffusion capacity, reduced lung volume, or airflow obstruction. Impaired diffusion capacity (DLCO < 80%) was the most common abnormality, observed in six studies (47%, 95% CI 32–61%), followed by reduced lung volume measurements, including TLC <80% (14%, 95% CI 9–18%), FVC <80% (12%, 95% CI 1–23%), and FEV1 <80% (7%, 95% CI 5–9%). Airflow obstruction (FEV1/FVC <70%) was relatively uncommon, and only reported in three studies (9%, 95% CI 0–18%).

### Risk of Post-acute Manifestations and Abnormal Lung Function in Severe Patients Compared to Non-severe Patients

**Figure 3:**
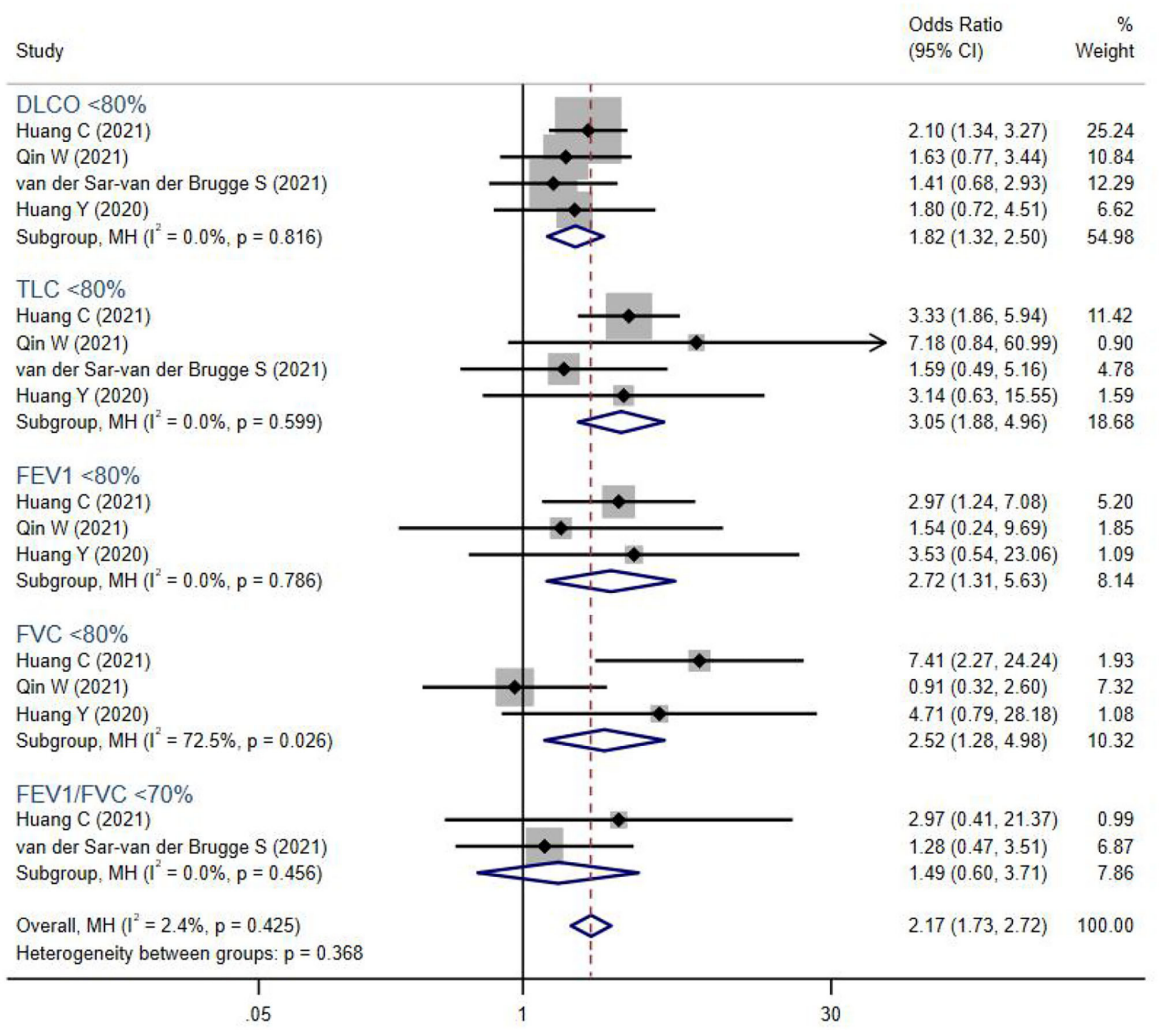
The forest plot of the odds ratio (OR) for risk of abnormal lung function parameters in severe patients compared to non-severe patients, with a range from 0.91 to 7.41. The brown dashed line at 2.17 on the x-axis represents the overall odds ratio (OR 2.17, 95% CI 1.73–2.72) for these parameters. The blue rhombuses that are centered to the right of the vertical solid line indicate that severe patients are more likely to develop lung function abnormalities in the post-acute phase. DLCO, diffusion capacity for carbon monoxide; TLC, total lung capacity; FEV1, forced expiratory volume in the first second; FVC, forced vital capacity.

We also compared the systemic symptoms and pulmonary functions between severe and non-severe COVID-19 patients. As shown in Table 3 and Figure 3, severe patients were more likely to develop adverse manifestations of the musculoskeletal (OR 1.60, 95% CI 1.12–2.29), cardiopulmonary (OR 1.36, 95% CI 1.13–1.64), and psychosocial (OR 1.23, 95% CI 1.02–1.48) systems. More specifically, joint pain (OR 1.84, 95% CI 1.11–3.04), dyspnea (OR 1.52, 95% CI 1.12–2.06), palpitation (OR 1.57, 95% CI 1.07–2.30), and anxiety or depression (OR 1.42, 95% CI 1.03–1.97) were more prevalent among severe COVID-19 cases than in non-severe subjects. The occurrence of fatigue or weakness, the most common symptoms of post-acute COVID-19 syndrome, were not significantly different between the two groups.

**Table 3:** The pooled OR of symptoms and manifestations in multiple systems between severe and non-severe patients.

**Outcomes**		**Studies**	**Sample sizes**			**Effects**	
			**Severe**	**Non-severe**	**OR (95% CI)**	***I*^**2**^**	***p* for heterogeneity**
Fatigue or weakness		5	218	1,901	1.13 (0.91, 1.42)	0.0%	0.884
Cardiopulmonary	Chest pain	5	434	2,232	1.09 (0.64, 1.83)	41.0%	0.148
	Dyspnea	5	349	762	1.52 (1.12, 2.06)	0.0%	0.903
	Cough	4	317	694	1.04 (0.65, 1.64)	38.2%	0.183
	Sore throat	3	176	1,713	1.37 (0.73, 2.56)	0.0%	0.661
	Palpitation	2	365	1,937	1.57 (1.07, 2.30)	16.4%	0.274
	Overall				1.36 (1.13, 1.64)	0.0%	0.610
Musculoskeletal	Myalgia	3	162	1,737	1.42 (0.85, 2,35)	0.0%	0.716
	Joint pain	2	135	1,630	1.84 (1.11, 3.04)	59.3%	0.117
	Overall				1.60 (1.12, 2.29)	0.0%	0.465
Neurological	Memory impairment	3	83	271	0.70 (0.40, 1.24)	0.0%	0.667
	Taste disorder	3	168	1,741	0.96 (0.52, 1.74)	0.0%	0.412
	Smell disorder	3	159	1,726	1.14 (0.70, 1.87)	0.0%	0.392
	Overall			111	0.93 (0.67, 1.28)	0.0%	0.630
Gastrointestinal	Decreased appetite	2	149	1,606	1.06 (0.58, 1.94)	0.0%	0.600
Psychosocial	Anxiety or depression	3	170	1,681	1.42 (1.03, 1.97)	17.6%	0.297
	Attention deficit disorder	2	56	164	1.18 (0.60, 2.30)	67.5%	0.080
	Sleep difficulties	4	186	1,833	1.14 (0.83, 1.56)	15.7%	0.313
	Hair loss	2	141	1,634	1.14 (0.77, 1.70)	0.0%	0.749
	Overall				1.23 (1.02, 1.48)	2.5%	0.418

In addition, severe COVID-19 patients were more likely to suffer from persistent abnormal pulmonary functions (OR 2.17, 95% CI 1.73–2.72). Lung volumes were significantly reduced in severe COVID-19 patients, including TLC <80% (OR 3.05, 95% CI 1.88–4.96), FEV1 <80% (OR 2.72, 95% CI 1.31–5.63), FVC <80% (OR 2.52, 95% CI 1.28–4.98), and DLCO <80% (OR 1.82, 95% CI 1.32–2.50). However, the PFT parameters FEV1/FVC <70%, which indicated airway obstruction, showed no difference between groups.

## Discussion

In the current study, we analyzed the data retrieved from 16 cohort studies with verified and hospitalized COVID-19 patients. Cumulatively, these studies reported a total of 29 multi-system symptoms associated with post-acute COVID-19, involving the cardiopulmonary (15%), neurological (15%), musculoskeletal (13%), gastrointestinal systems (7%), and psychosocial manifestations (28%). The most common symptoms were fatigue or weakness (47%), memory impairment (35%), anxiety or depression (33%), and dyspnea (33%), while DLCO < 80% (47%) was widely present among these post-acute phase patients. Patients who recovered from severe COVID-19 were more likely to develop joint pain (OR 1.84), dyspnea (OR 1.52), palpitation (OR 1.57), and anxiety or depression (OR 1.42). Moreover, PFT parameters were all significantly different between severity groups. Collectively, these residual multi-system manifestations illustrate that hospital discharge does not indicate complete recovery, and that these patients require prolonged care, ideally through multidisciplinary clinics capable of comprehensive rehabilitation strategies.

Lopez et al. (3) reported more than 50 long-term symptoms of post-COVID-19 syndrome, among which fatigue (58%) was the most common, in agreement with our findings of a high prevalence of fatigue or weakness (47%). Previous work by Perrin and co-workers suggested that a subset of COVID-19 patients may develop chronic fatigue syndrome (CFS) similar to those reported to follow SARS and MERS (32). CFS is characterized by persistent or recurrent unexplained severe fatigue that is not improved by rest, and may be accompanied by manifestations such as myalgia, depression, and sleep disorder. Perrin and colleagues also recently proposed that CFS could be induced by SARS-CoV-2 invasion of olfactory neurons, which can result in congestion of the lymphatic duct and subsequent accumulation of toxic agents in the central nervous system (33), thus induction of lymphatic circulation might be an effective measure to alleviate CFS follow COVID-19.

Moreover, it was noteworthy that there was a significantly higher prevalence of psychosocial manifestations (28%) compared with the cardiopulmonary, neurological, musculoskeletal, and gastrointestinal systems. Patients previously infected with other coronaviruses also presented with serious depression, anxiety, and PTSD (13). This finding suggested that the COVID-19 pandemic may have caused more profound impacts on mental health than direct organ damage. For such psychosocial disorders, the effectiveness of a one-size-fits-all response can vary greatly between different groups (34). Thus, epidemiological studies are warranted to evaluate the long-term impacts on mental health and social function in overall and specific groups of COVID-19 patients (such as the elderly, children, patients with mental complications, patients with different income levels, etc.) (35).

In addition to the psychological effects, a large body of evidence indicates that the lung is the most severely affected organ in COVID-19 patients (36). The related histopathological findings include diffuse alveolar epithelial injury, capillary injury or hemorrhage, hyaline membrane formation, fibrous hyperplasia of the alveolar septum, and lung consolidation (37). These cumulative changes could generate markedly adverse impacts on respiratory capacity in COVID-19 patients, but it is not clear whether lung functions remain impaired throughout the post-acute phase. In a previous meta-analysis conducted by Torres et al. (38), DLCO (39%) impairment was the most commonly observed lung function abnormality in post-infection COVID-19 patients, which agreed with our results of 47% prevalence of DLCO <80%. The higher prevalence is potentially due to our inclusion of only hospitalized patients who suffered relatively severe disease symptoms. More importantly, this result suggested the pulmonary diffusion capacity of recovering patients remained extensively affected through 1 month post-discharge or 2 months post-admission, and might largely explain the persistence of residual respiratory symptoms such as dyspnea (33%), since physical function tests are typically more reliable than self-reported symptoms in reflecting a patient's actual health status. The decrease in DLCO is often associated with pulmonary fibrosis, such as interstitial disease and systemic sclerosis (39, 40). Cases of pulmonary fibrosis have been reported in COVID-19 recovery patients (41), and thus long-term follow-ups are necessary to determine whether DLCO damage in post-acute patients indicates an increased risk of pulmonary fibrosis.

One cohort study (25) reported that ICU care was a risk factor for impaired lung function. Our results were consistent with this finding, and also showed differences in TLC, FEV1, FVC, and DLCO abnormalities between severe and non-severe post-acute COVID-19 patients, among which TLC <80% (OR 3.05) was the most significant. Although we found no difference in FEV1/FVC < 70% between severity groups, its related parameters FEV1 and FVC were both lower and more likely to be abnormal in severe patients. This suggested the higher severity was associated with more seriously impaired diffusion capacity, as well as restrictive and obstructive dysfunction in post-acute patients. A previous study (42) in SARS implied that pulmonary function could be improved when viral pneumonia was effectively managed at the acute phase, whereas almost no substantial recovery was observed in the following 2 years after infection. Consequently, once the infection is controlled, exercise programs should be undertaken as early as possible to strengthen lung function in severe patients. It is also important to note that this study did not include pre-existing respiratory diseases which contribute to abnormal lung function, possibly leading to an overestimation of the effects of COVID-19 alone.

Lastly, we also compared events of post-acute symptoms in discharged COVID-19 patients with severe and non-severe diseases COVID-19 patients. Generally, there was no significant difference in overall symptoms of multiple organs or systems between the two groups with different levels of severity, with only just slight differences in musculoskeletal (OR 1.60), cardiopulmonary (OR 1.36), and psychosocial (OR 1.23) manifestations. Among musculoskeletal symptoms, however, it was noteworthy that joint pain (OR 1.84) was more likely to appear in severe patients than the non-severe. In our previous follow-up study (42) in SARS patients, joint disorders persisting over 15 years were presumed to be closely related to high-dose steroid therapy and had little direct relation with the viral infection. As systemic glucocorticoids were recommended for use in hospitalized COVID-19 patients who require mechanical ventilation, regular assessments of joint function and prophylactically rehabilitative interventions of musculoskeletal systems should be advocated for these severe populations.

This meta-analysis also had limitations that should be addressed in future and ongoing studies. First, quality assessment of the included cohort studies revealed that most studies were of low to medium quality. Second, we observed high heterogeneity in the prevalence of both symptoms and lung function, possibly due to wide variation in follow-up duration (1–6 months), disease severity, and sample sizes (55–1,733 patients). The method of self-reporting symptoms through questionnaires could also lead to bias, so it is necessary to standardize investigational methods for post-acute COVID-19 syndrome, such as through the adoption of the “Post-COVID-19 Functional Status Scale” proposed by Klok et al. (43). Third, not all outcomes were examined through subgroup analysis because of an insufficient number of studies and too many outcome indicators. In particular, subgroup analysis based on treatment regimen during hospitalization was not possible based on a lack of information, though future studies exploring this point will likely reveal important differences in therapeutic approach that affect the long-term symptoms. The available treatment information for each study is provided in Supplementary Table 2. Given that COVID-19 is a newly-emerged epidemic, longer follow-up studies are recommended to explore the effects of age, pre-existing disease, duration, and certain interventions on post-acute COVID-19 syndrome, especially from the functional parameters of multiple systems.

## Conclusion

The evidence presented in this systematic review and meta-analysis supports the wide spectrum of multi-system manifestations associated with post-acute COVID-19. Fatigue or weakness, progressing to CFS, was the most prevalent physiological symptom, and although respiratory dysfunction is widespread among discharged patients, especially diffusion disorders, pandemic-related psychosocial effects were more extensive in survivors than direct physical damage by SARS-CoV-2. In addition, we found that severe COVID-19 is a risk factor for abnormalities in almost all PFT parameters, and that exercise regimens adopted soon after the acute phase, with regular assessments of lung and joint function, could potentially alleviate long-term COVID-19 symptoms.

## Data Availability Statement

The original contributions presented in the study are included in the article/Supplementary Material, further inquiries can be directed to the corresponding author/s.

## Author Contributions

QL designed the analysis and wrote the first draft. XH and YB collected the data. JL analyzed the data. YZ and ZG supervised the work and edited the final version of the paper. All authors contributed to the article and approved the submitted version.

## Conflict of Interest

The authors declare that the research was conducted in the absence of any commercial or financial relationships that could be construed as a potential conflict of interest.

## Publisher's Note

All claims expressed in this article are solely those of the authors and do not necessarily represent those of their affiliated organizations, or those of the publisher, the editors and the reviewers. Any product that may be evaluated in this article, or claim that may be made by its manufacturer, is not guaranteed or endorsed by the publisher.
