# Supplementary material for: Follow-Ups on Persistent Symptoms and Pulmonary Function Among Post-Acute COVID-19 Patients: A Systematic Review and Meta-Analysis
Source: Front Med (Lausanne). 2021 Sep 3;8:702635. doi: 10.3389/fmed.2021.702635 (PMC8448290; doi:10.3389/fmed.2021.702635)
Supplement: Supplementary file 1 [file Data_Sheet_1.docx]

**
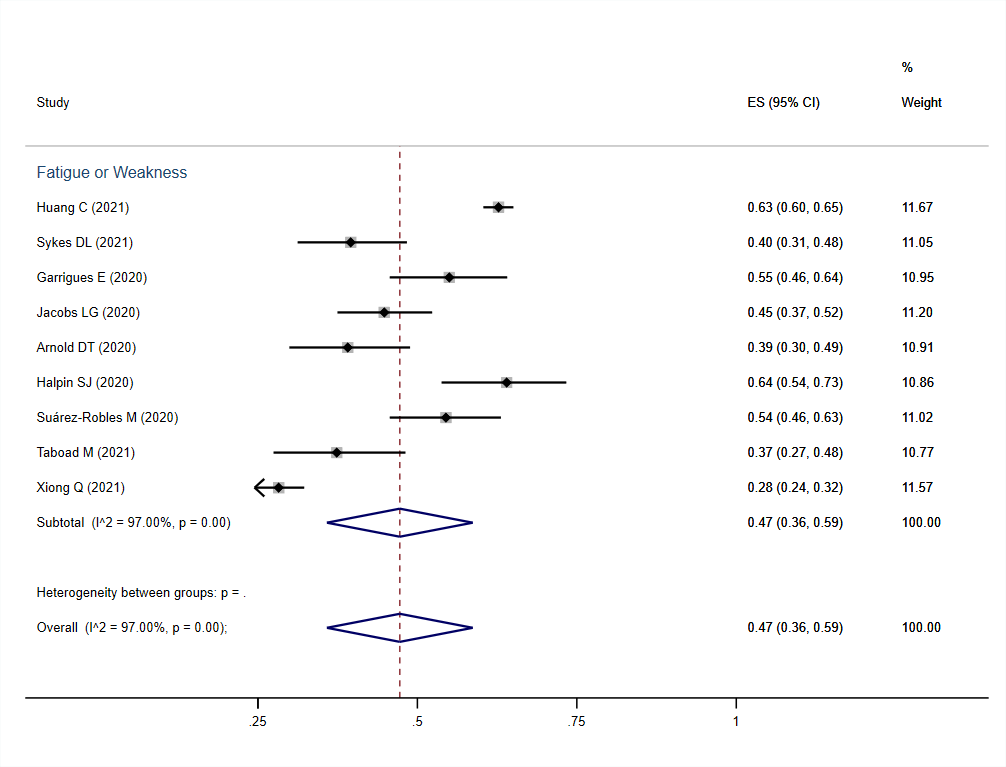
**

**Supplementary Figure 1.** The forest plot for prevalence of fatigue or weakness symptom.


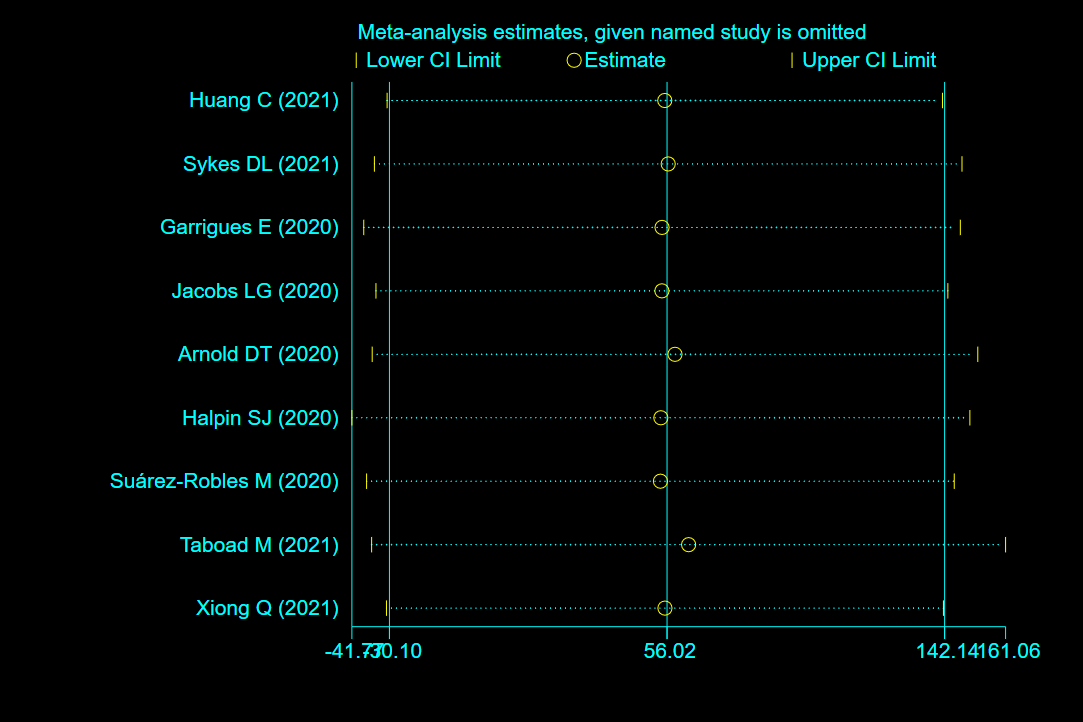


**Supplementary Figure 2**. The sensitivity analysis for prevalence of fatigue or weakness.

**
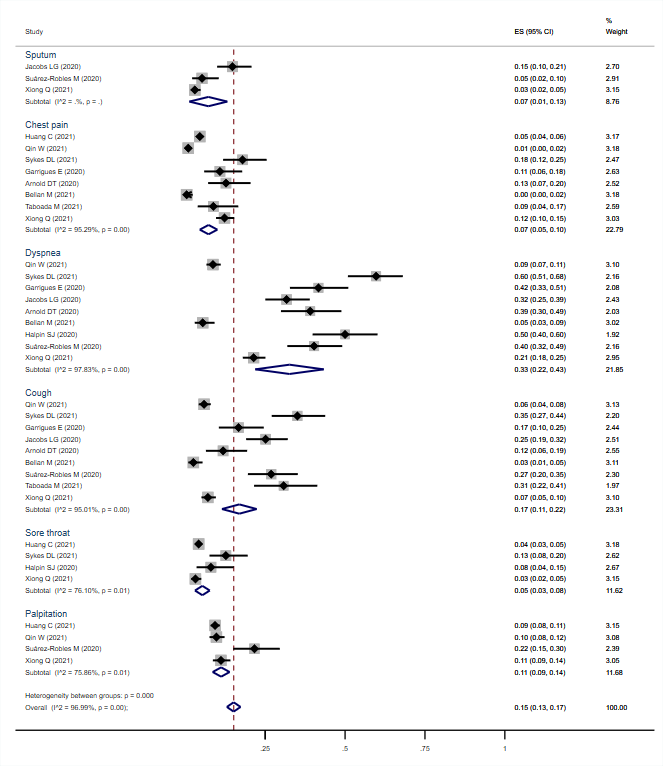
**

**Supplementary Figure 3.** The forest plot for prevalence of cardiopulmonary manifestations.


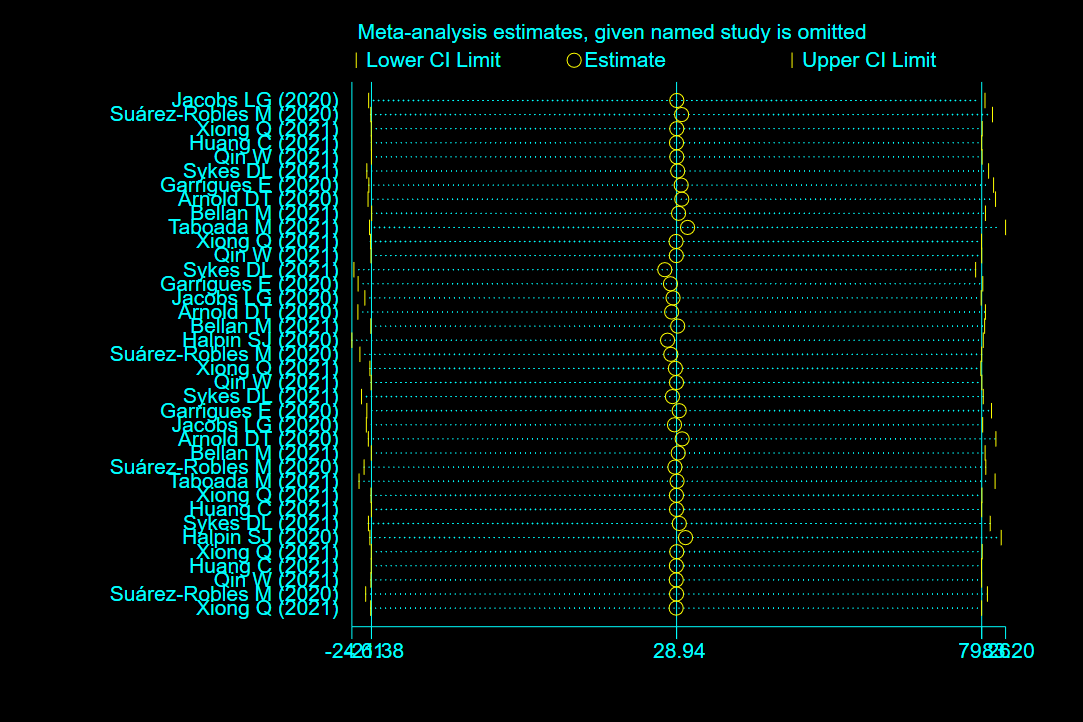


**Supplementary Figure 4**. The sensitivity analysis for prevalence of cardiopulmonary manifestations.


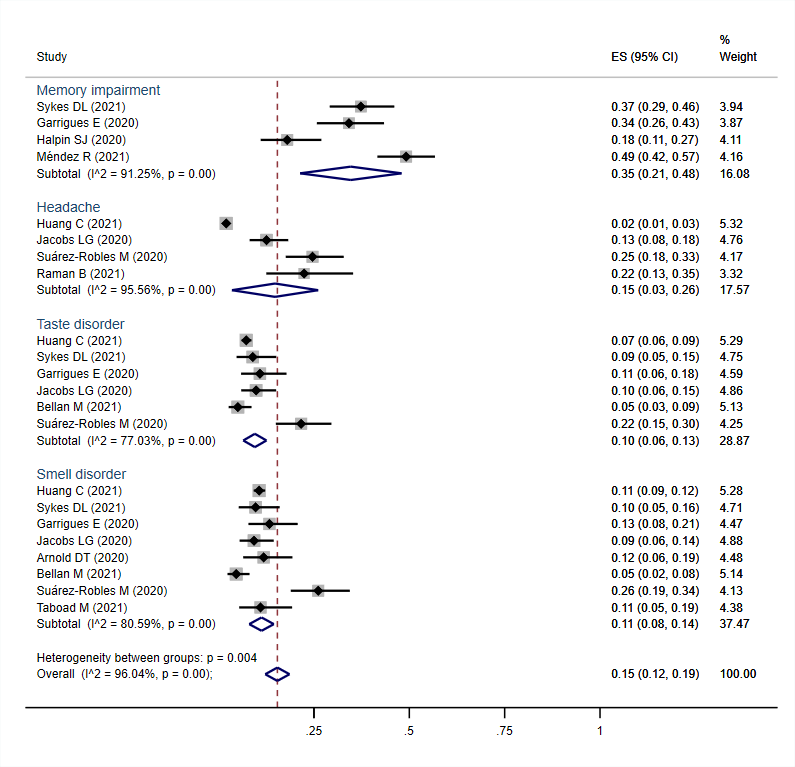


**Supplementary Figure 5.** The forest plot for prevalence of neurological manifestations.


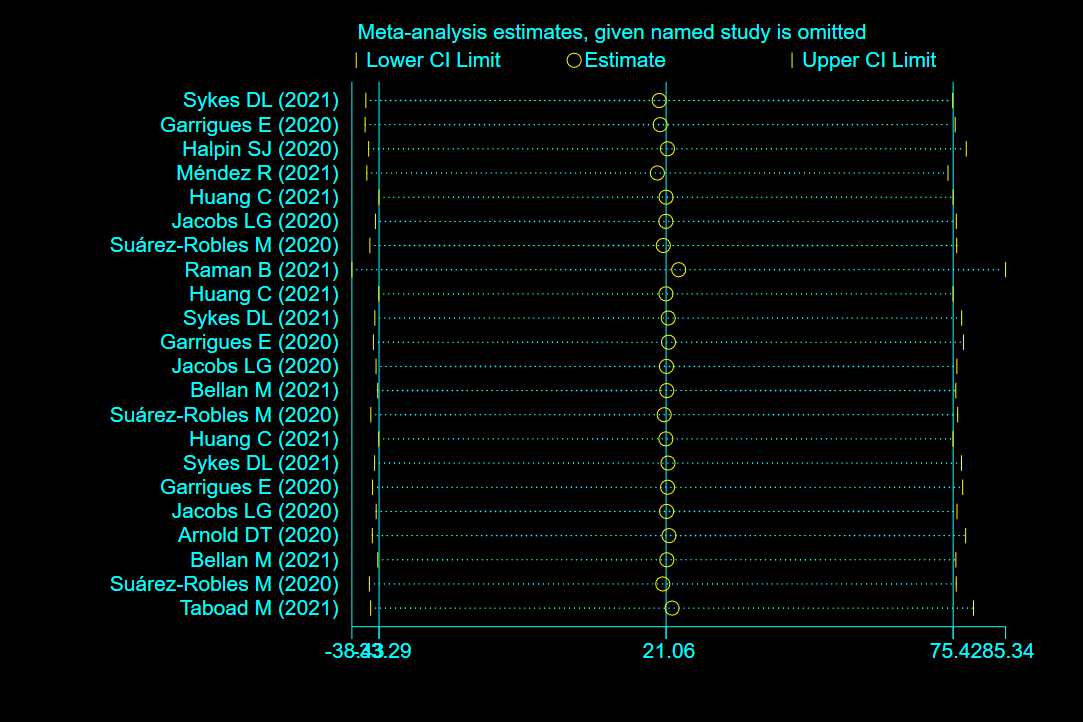


**Supplementary Figure 6**. The sensitivity analysis for prevalence of neurological manifestations.


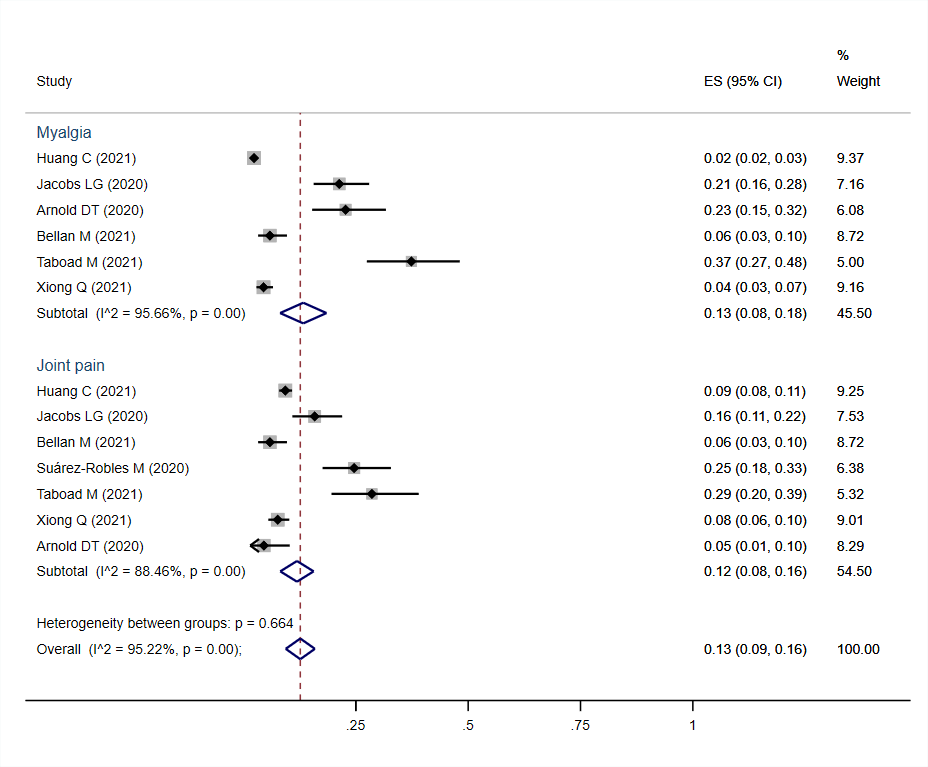


**Supplementary Figure 7.** The forest plot for prevalence of musculoskeletal manifestations.


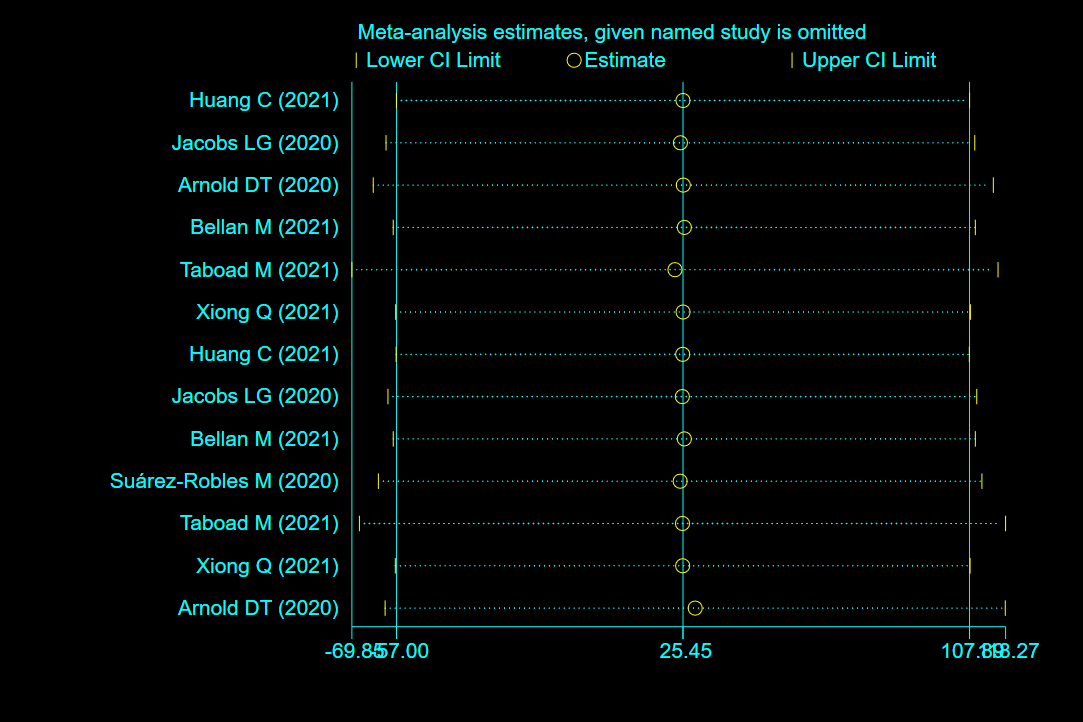


**Supplementary Figure 8**. The sensitivity analysis for prevalence of musculoskeletal manifestations.


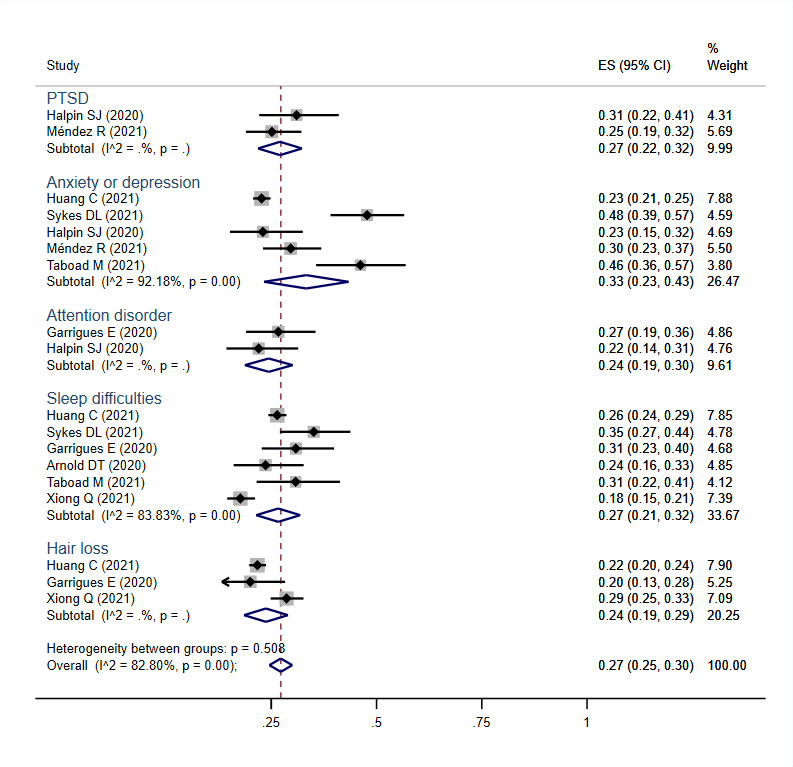


**Supplementary Figure 9.** The forest plot for prevalence of psychosocial manifestations.


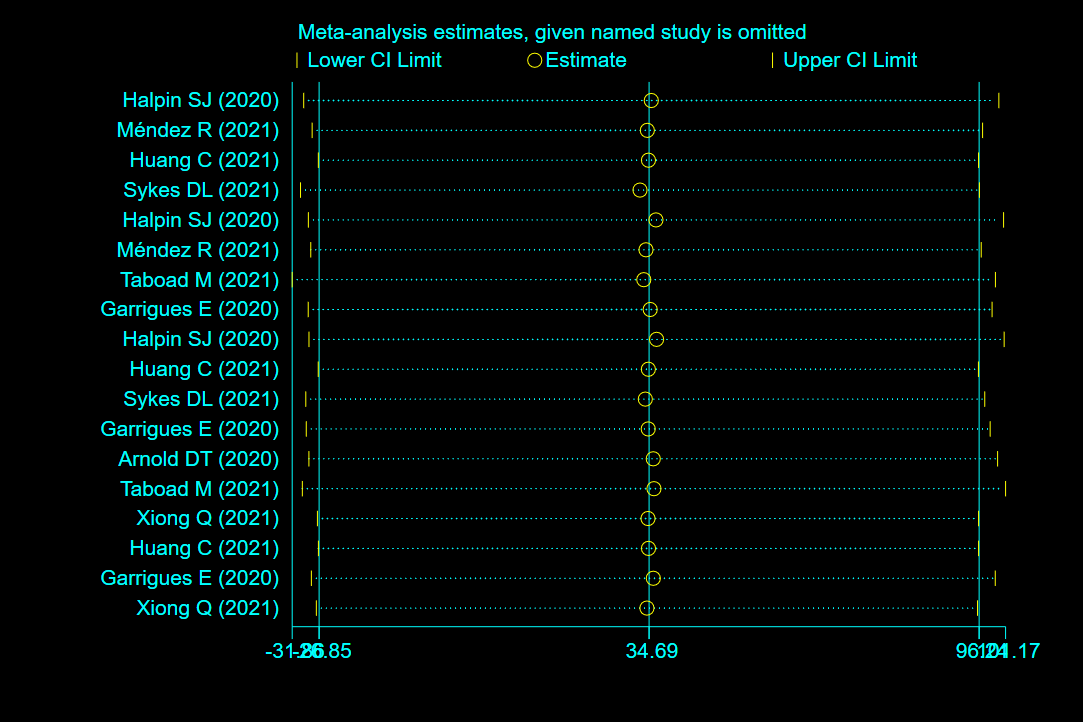


**Supplementary Figure 10**. The sensitivity analysis for prevalence of psychosocial manifestations.

**
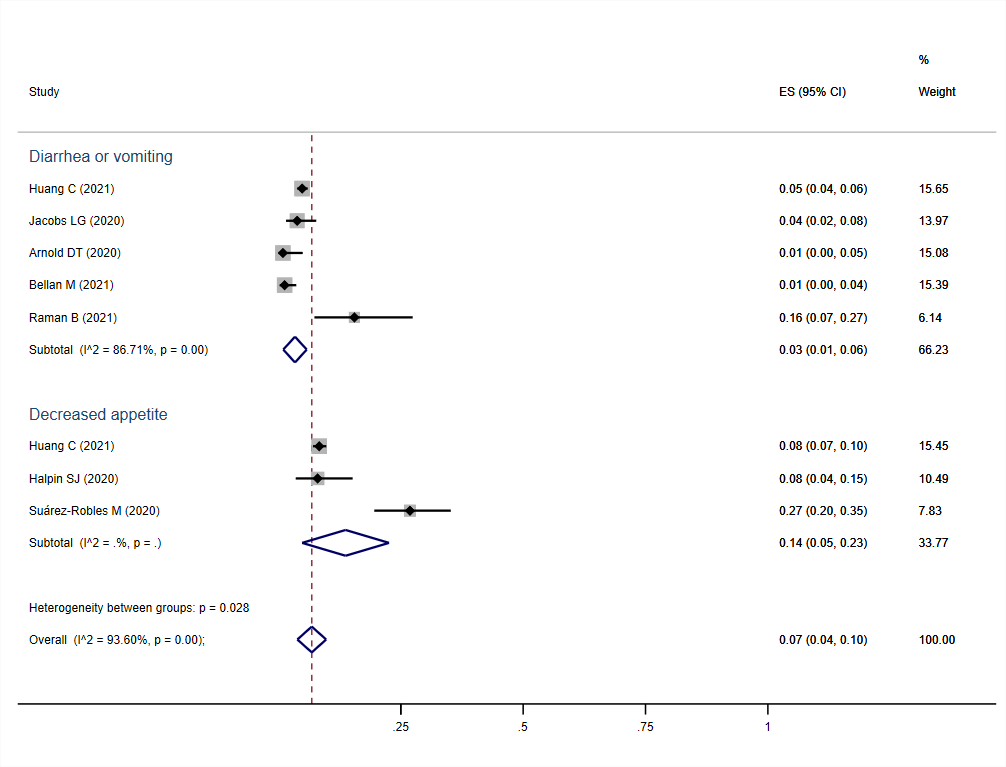
**

**Supplementary Figure 11.** The forest plot for prevalence of gastrointestinal manifestations.


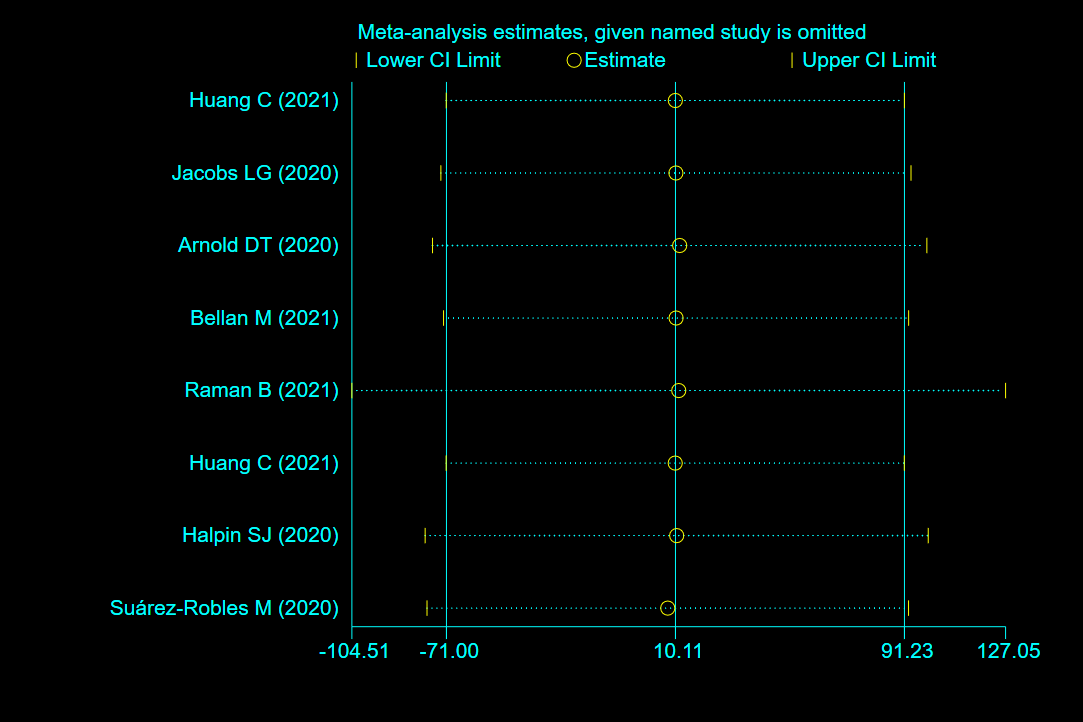


**Supplementary Figure 12**. The sensitivity analysis for prevalence of gastrointestinal manifestations.

**
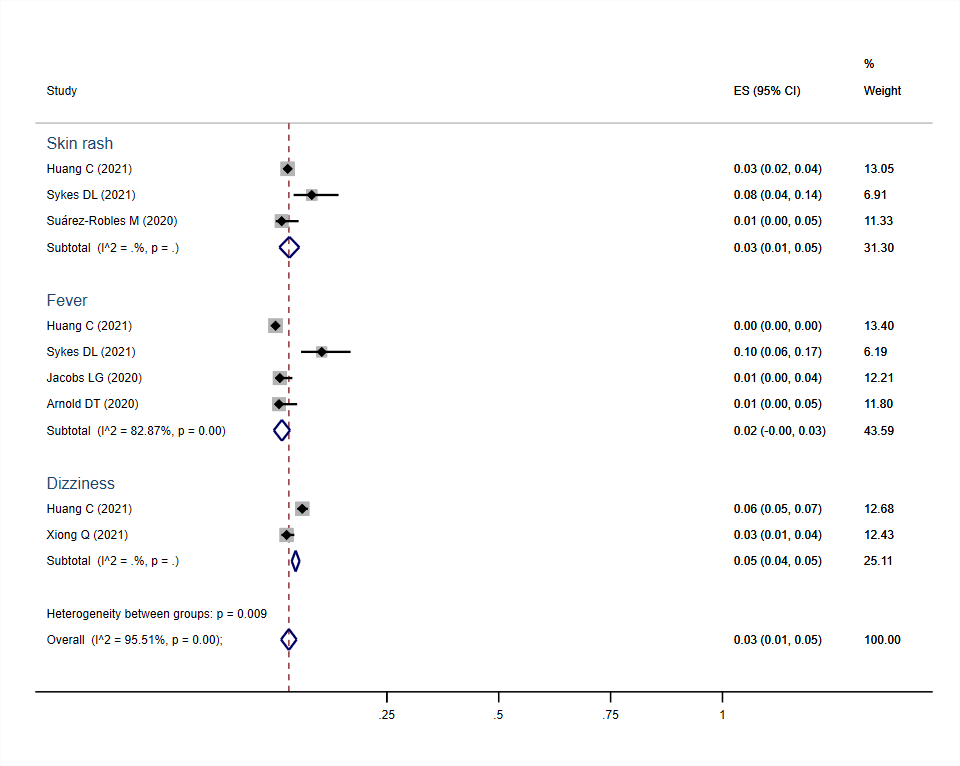
**

**Supplementary Figure 13.** The forest plot for prevalence of other symptoms and manifestations.


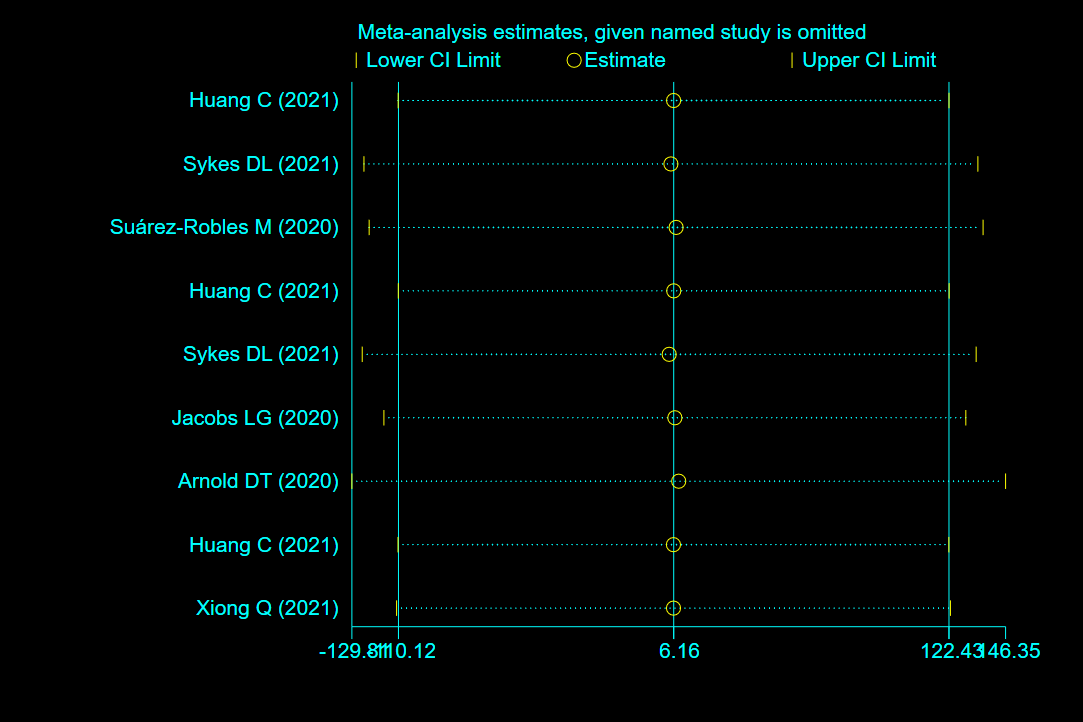


**Supplementary Figure 14**. The sensitivity analysis for prevalence of other symptoms and manifestations.


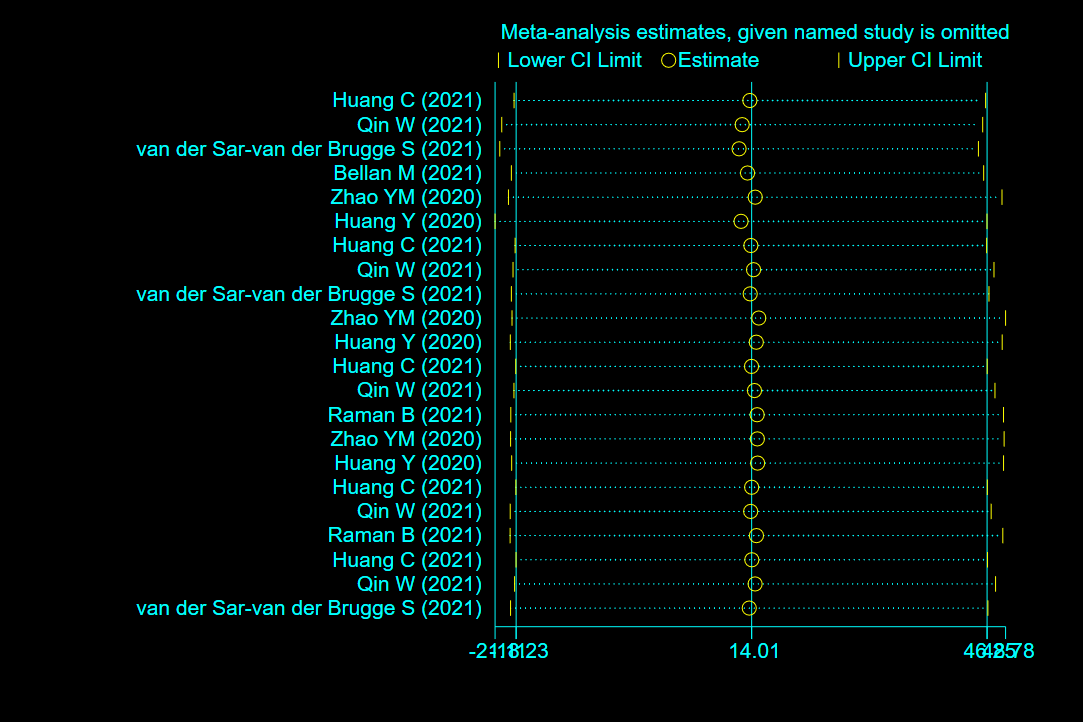


**Supplementary Figure 15**. The sensitivity analysis for prevalence of abnormal lung function parameters.


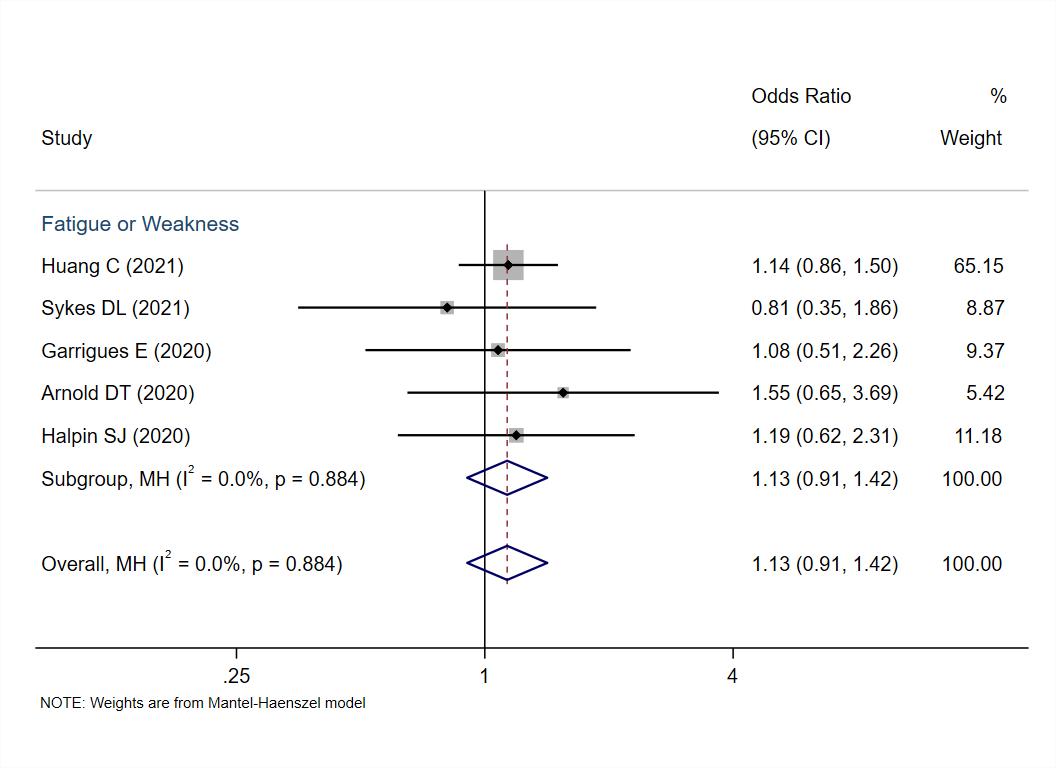


**Supplementary Figure 16**. The forest plot for risk of fatigue or weakness symptom in severe patients compared to non-severe patients.


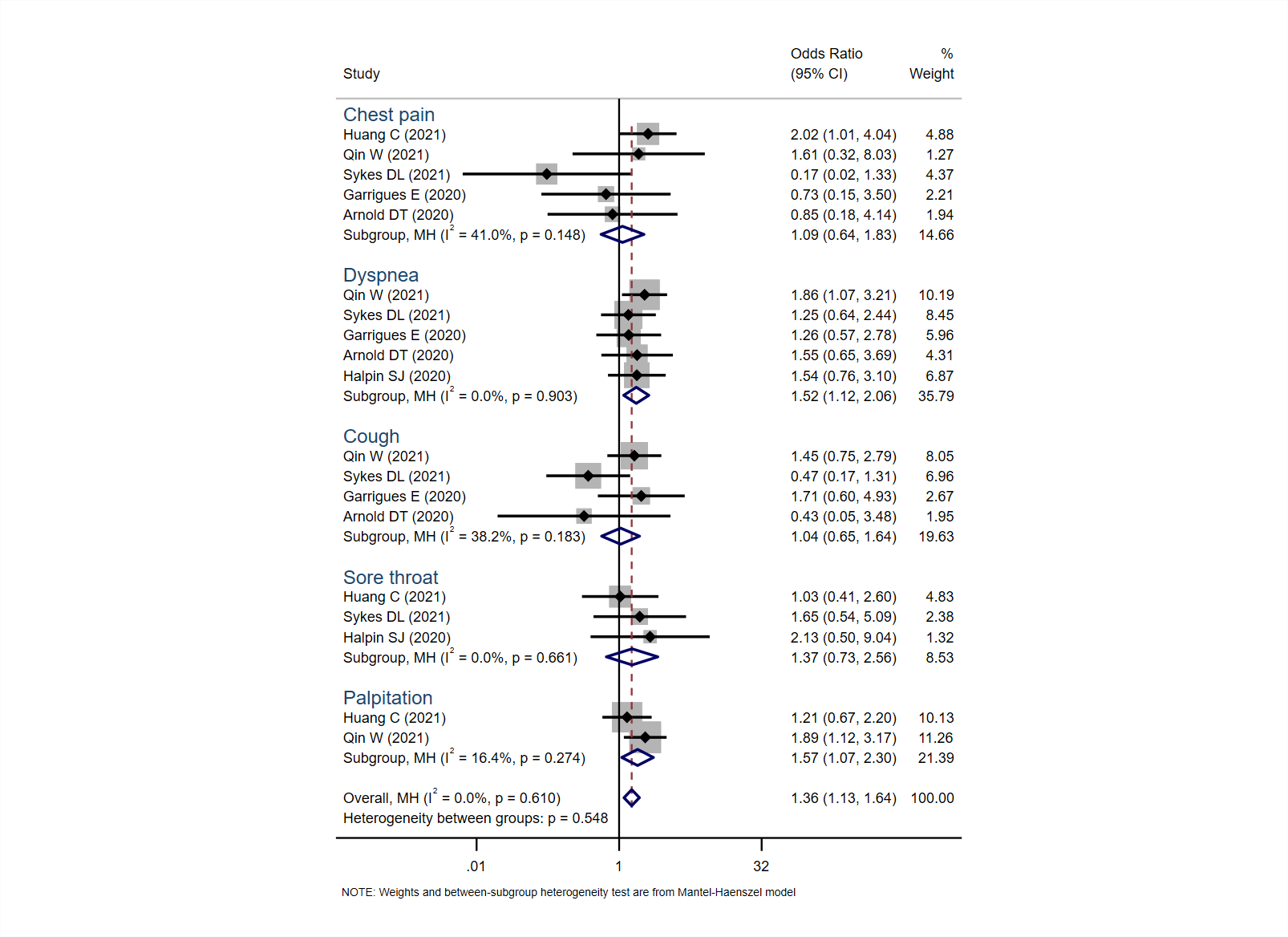
**Supplementary Figure 17**. The forest plot for risk of cardiopulmonary manifestations in severe patients compared to non-severe patients.


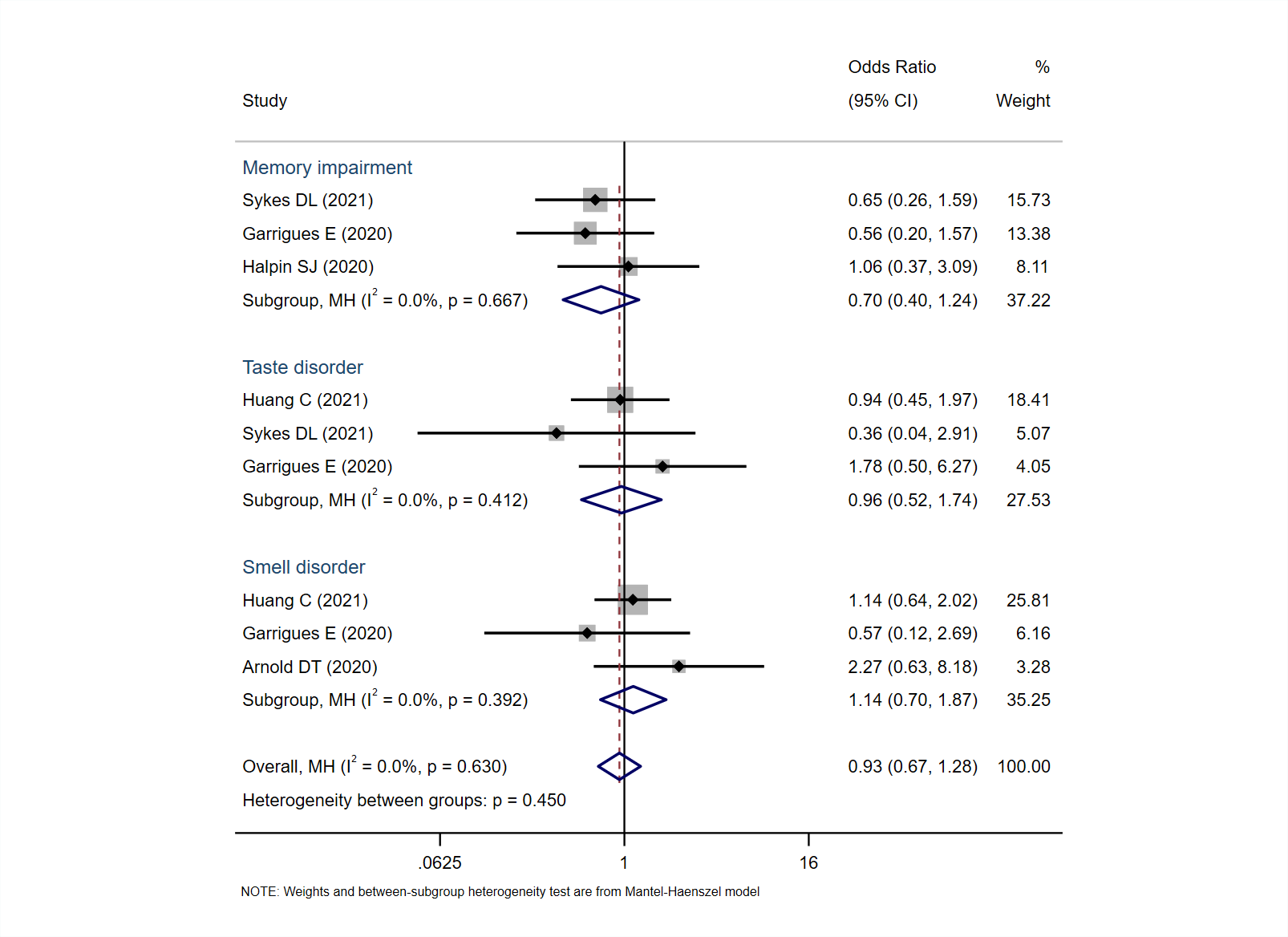


**Supplementary Figure 18**. The forest plot for risk of neurological manifestations in severe patients compared to non-severe patients.


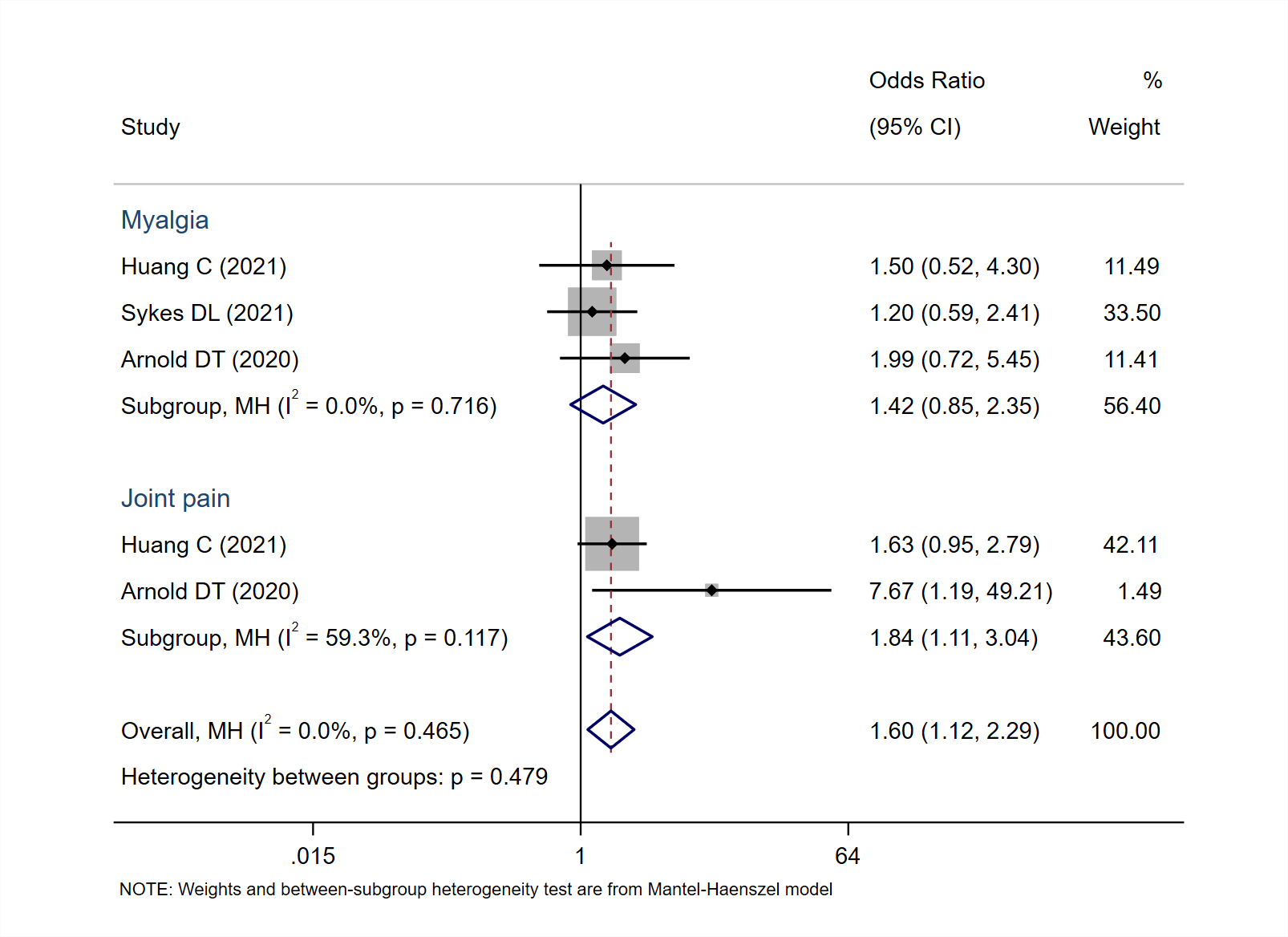


**Supplementary Figure 19**. The forest plot for risk of musculoskeletal manifestations in severe patients compared to non-severe patients.


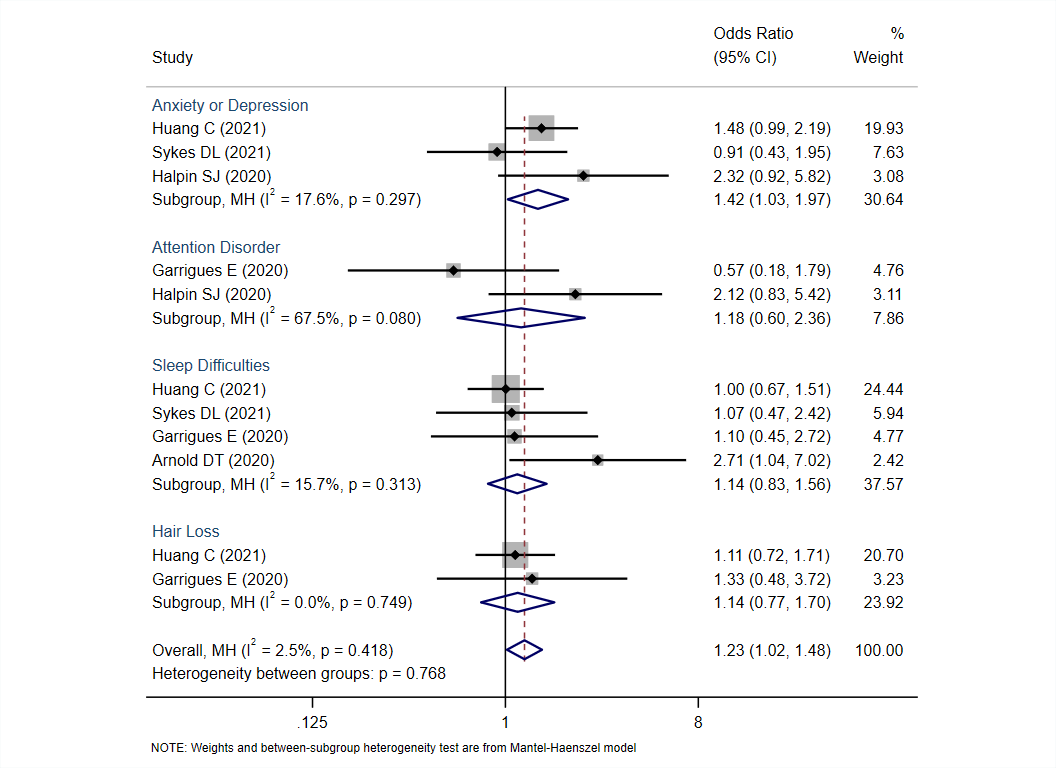


**Supplementary Figure 20**. The forest plot for risk of psychological manifestations in severe patients compared to non-severe patients.


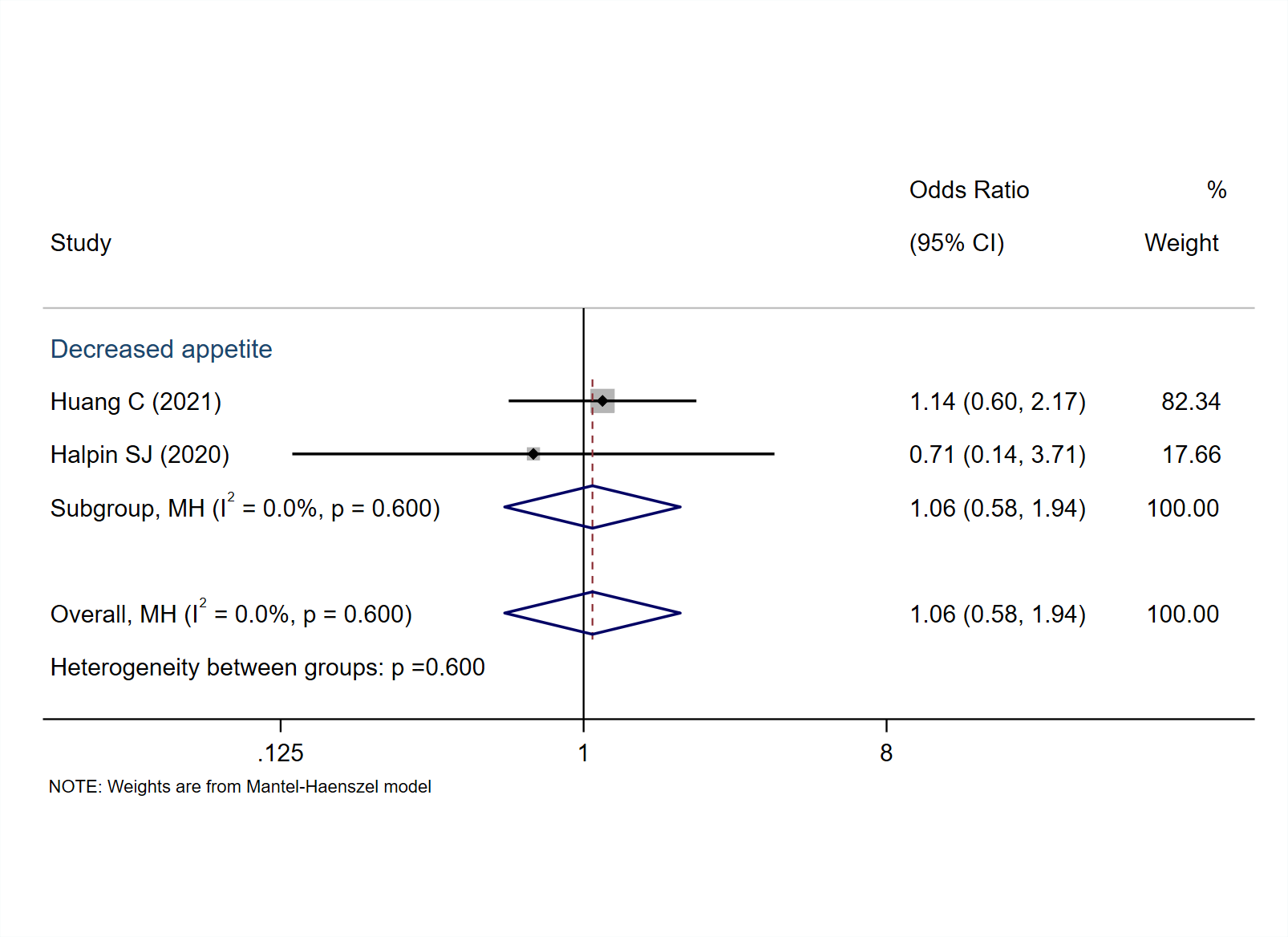


**Supplementary Figure 21**. The forest plot for risk of gastrointestinal manifestations in severe patients compared to non-severe patients.
