# Supplementary material for: Follow-Ups on Persistent Symptoms and Pulmonary Function Among Post-Acute COVID-19 Patients: A Systematic Review and Meta-Analysis
Source: Front Med (Lausanne). 2021 Sep 3;8:702635. doi: 10.3389/fmed.2021.702635 (PMC8448290; doi:10.3389/fmed.2021.702635)
Supplement: Supplementary file 2 [file Data_Sheet_2.docx]

**Table S1.** Outcomes of included studies in the meta-analysis.

| **Author** | **Outcomes** | | | | | | | |
| --- | --- | --- | --- | --- | --- | --- | --- | --- |
|  | **Fatigue** | **Cardiopulmonary** | **Gastrointestinal** | **Musculoskeletal** | **Psychosocial** | **Neurological** | **Others** | **Lung function** |
| Huang C et al. | Fatigue or weakness | Chest pain，Sore throat，Palpitation | Diarrhea or vomiting，Decreased appetite | Myalgia，Joint pain | Anxiety or depression，Sleep difficulties，Hair loss | Headache，Taste disorder，Smell disorder | Skin rash，  Fever，  Dizziness  Pain or discomfort | DLCO<80%，TLC<80%，FEV1<80%，FVC<80%，FEV1/FVC<70% |
| Qin W et al. | - | Chest pain，Dyspnea，Cough，Palpitation | - | - | - | - | - | DLCO<80%，TLC<80%，FEV1<80%，FVC<80%，FEV1/FVC<70% |
| Sykes DL et al. | Fatigue or weakness | Chest pain，Dyspnea，Cough，Sore throat | - | - | Anxiety or depression，Sleep difficulties | Memory impairment，Taste disorder，Smell disorder | Skin rash，Fever | - |
| Garrigues E et al. | Fatigue or weakness | Chest pain，Dyspnea，Cough | - | - | Attention disorder，Sleep difficulties，Hair loss | Memory impairment，Taste disorder，Smell disorder | - | - |
| van der Sar-van der Brugge S et al. | - | - | - | - | - | - | - | DLCO<80%，TLC<80%，FEV1/FVC<70% |
| Jacobs LG et al. | Fatigue or weakness | Sputum，Chest pain，Cough | Diarrhea or vomiting | Myalgia，Joint pain | - | Headache，Taste disorder，Smell disorder | Fever，Eye problems | - |
| Arnold DT et al. | Fatigue or weakness | Chest pain，Dyspnea，Cough | Diarrhea or vomiting，Abdominal pain | Myalgia，Joint pain | Sleep difficulties | Smell disorder | Fever | - |
| Bellan M et al. | - | Chest pain，Dyspnea，Cough | Diarrhea or vomiting | Myalgia，Joint pain | - | Taste disorder，Smell disorder | - | DLCO<80% |
| Halpin SJ et al. | - | Dyspnea，Sore throat | Decreased appetite | - | PTSD，Anxiety or depression，Attention disorder | Memory impairment | - | - |
| Suárez-Robles M et al. | Fatigue or weakness | Sputum，Dyspnea，Cough，Palpitation | Decreased appetite | Joint pain | - | Headache，Taste disorder，Smell disorder，Sensitivity disorders | Skin rash | - |
| Méndez R et al. | - | - | - | - | PTSD，Anxiety or depression | Memory impairment，Cognitive impairment | - | - |
| Raman B et al. | - | - | Diarrhea or vomiting | - | - | Headache | - | FEV1<80%，FVC<80% |
| Taboada M et al. | Fatigue or weakness | Chest pain | - | Myalgia，Joint pain | Anxiety or depression，Sleep difficulties | Smell disorder | - | - |
| Xiong Q et al. | Fatigue or weakness | Sputum，Chest pain，Dyspnea，Cough，Sore throat，Palpitation， Chest distress | - | Myalgia，Joint pain | Sleep difficulties，Hair loss | - | Dizziness，Sweeting，Chills | - |
| Zhao YM et al. | - | - |  | -- | - | - | - | DLCO<80%，TLC<80%，FEV1<80% |
| Huang Y et al. | - | - | - | - | - | - | - | DLCO<80%，TLC<80%，FEV1<80% |

**Table S2.** The grouping of original data in included studies.

| **Author** | **Sample Sizes** | **Grouping method** | **Numbers in each group** | **Subgroup analysis** | **Numbers in each subgroup** |
| --- | --- | --- | --- | --- | --- |
| Huang C et al. | 1733 | WHO severity scale | Scale 3 (439)  Scale 4 (1172)  Scale 5–6 (122) | - | - |
| Qin W et al. | 647 | Disease severity | Non-severe (399)  Severe (248) | Lung function at  three-month follow-up | Normal DLCO (37)  Impaired DLCO (44) |
| Sykes DL et al. | 134 | Departments of admissions | Ward-based (107)  ICU (27) | Gender | Male (88)  Female (46) |
|  |  |  |  | Follow-up period | Follow-up 47–75 Days (7)  Follow-up 76–100 Days (26)  Follow-up 101–125 Days (78)  Follow-up 126–167 Days (23) |
| Garrigues E et al. | 120 | Departments of admissions | Ward (96)  ICU (24) | - | - |
| van der Sar-van der Brugge S et al. | 101 | WHO severity scale | Scale 3 (28)  Scale 4 (73) | - | - |
| Jacobs LG et al. | 183 | Persisting symptoms experience | Ever experienced  Not experienced | - | - |
| Arnold DT et al. | 110 | Disease severity | Mild (27)  Moderate (65)  Severe (18) | - | - |
| Bellan M et al. | 238 | - | - | - | - |
| Halpin SJ et al. | 100 | Departments of admissions | Ward (68)  ICU (32) | - | - |
| Suárez-Robles M et al. | 134 | - | - | - | - |
| Méndez R et al. | 179 | - | - | - | - |
| Raman B et al. | 58 | - | - | - | - |
| Taboada M et al. | 91 | - | - | - | - |
| Xiong Q et al. | 538 | Disease severity | General (331)  Severe (180)  Critical (27) | Complications | Physical decline/fatigue  Yes (152) No(386) |
|  |  |  |  |  | Postactivity polypnoea  Yes (115) No (423) |
|  |  |  |  |  | Resting heart rate increase  Yes (60) No (478) |
|  |  |  |  |  | Alopecia  Yes (154) No (384) |
| Zhao YM et al. | 55 | Lung function at  three-month follow-up | DLCO normal group (46)  DLCO impaired group (9) |  |  |
| Huang Y et al. | 57 | Disease severity | Severe (17)  Non-severe (40) | Prescription of GC | GC group (16)  Regular group (41) |

Abbreviations: ICU: intensive care unit, DLCO: diffusion capacity for carbon monoxide, GC: glucocorticoid.
